# Effect of Graphene Oxide on the Crystallization of Calcium Carbonate by C_3_S Carbonation

**DOI:** 10.3390/ma12132045

**Published:** 2019-06-26

**Authors:** Dapeng Zheng, Haibin Yang, Feng Yu, Bo Zhang, Hongzhi Cui

**Affiliations:** 1 College of Civil and Transportation Engineering, Shenzhen University, Shenzhen 518060, China; 2 Department of Architecture and Civil Engineering, City University of Hong Kong, Hong Kong, China

**Keywords:** calcium carbonate, graphene oxide, carbonization, polymorph, crystal size

## Abstract

The effect of graphene oxide (GO) on the crystallization of calcium carbonate (CaCO_3_) is explored in this paper. Precipitation of CaCO_3_ was carried out by bubbling carbon dioxide (CO_2_) through tricalcium silicate (C_3_S) hydration solution with different graphene oxide admixture contents (0.2%, 1% and 2% mass ratios based on C_3_S). The polymorph, morphology, crystal size and particle size of CaCO_3_ were evaluated using X-ray diffraction (XRD), an environmental scanning electronic microscope (ESEM), and laser particle size analysis. The results showed that addition of GO was able to promote the conversion of CaCO_3_ to a calcite crystal phase with higher thermal stability and crystallinity than the control. However, as the dosage of GO increased, the growth of the calcite crystal particles was somewhat suppressed, resulting in a decrease in the crystal particle size and a narrow particle size distribution. When the amount of GO was 0.2%, 1% and 2%, the crystal size of the calcite was 5.49%, 12.38%, and 24.61% lower than that of the sample without GO, respectively, while the particle size of the calcite also decreased by 17.21%, 39.26%, 58.03%, respectively.

## 1. Introduction

In recent years, the application of graphene oxide (GO) has been widely studied because of its unique physicochemical and structural properties [1,2]. Some studies [3,4,5,6,7,8] have reported that GO, as a reinforcing material, can improve the mechanical properties and microstructure of cementitious composites because of its high specific strength, high toughness, large specific surface area and low weight. Calcium carbonate (CaCO_3_), as a common cementitious composite, may have its carbonation process enhanced by applying nanomaterials such as GO. CaCO_3_ has been widely used in various industries, such as rubber, ink, plastics and building materials [9,10]. At present, various methods for preparing CaCO_3_ have been explored, such as carbonization, chemical precipitation [11], ultrasonic assisted synthesis [12], in situ deposition methods [13], and biologically controlled CaCO_3_ deposition [14,15]. Carbonization is an industrially useful method because it is environmentally friendly effectively uses mineral resources [16,17]. The crystallization, morphology, crystal size, and phase structure of CaCO_3_ are affected by admixture, temperature, concentration, and so on [18,19,20,21,22]. Currently, researchers usually use a variety of additives, such as surfactants, metallic ions, and GO, to control the crystallization, nucleation and crystal growth process of CaCO_3_ [23,24,25,26,27,28].

Carbonization is a very common phenomenon in the hydration process of cement. As the main product of the cement carbonization reaction, the formation of CaCO_3_ reduces the alkalinity of concrete and weakens the protection of steel bars [29]. On the other hand, the formation of CaCO_3_ can make concrete structures more compact and consequently improve their mechanical properties [30,31,32,33,34]. Besides, many studies have shown that calcium carbonates with different morphologies and crystalline structures have different effects on the microstructure of cement [34,35,36]. Due to the excellent mechanical properties of GO, the possibility of its application in cement has been extensively explored. Previous studies have reported that addition of GO promoted the hydration of cement and significantly increased the compressive and flexural strength of cement [37,38,39]. In a recent study, Lv et al. [40] found that the addition of GO was capable of affecting the shape of cement hydration products, such as ettringite (AFt) and monosulfate (AFm). However, Cui et al. [41] subsequently questioned this finding and experimentally confirmed that the change in shape of the hydration product was the result of a carbonization reaction. Nevertheless, in the carbonization process of cement, the effect of GO on the polymorph, morphology, and crystal size of CaCO_3_ has not been directly studied. 

Whether as an additive material or a carbonized product of cement, the properties of CaCO_3_ can have a significant impact on the performance of cement. As the major mineral component in Portland cement, the hydration reaction of tricalcium silicate (C_3_S) largely represents the hydration process of cement. Therefore, this study only focused on C_3_S hydration to simulate the hydration process of cement. Precipitation of CaCO_3_ was carried out by bubbling carbon dioxide (CO_2_) through C_3_S hydration solution with different GO admixture contents (0.2%, 1% and 2% mass ratios based on C_3_S). The effects of GO on the polymorphism, morphology, crystal size and particle size of CaCO_3_ during cement carbonization were investigated by X-ray diffraction (XRD), an environmental scanning electronic microscope (ESEM), and laser particle size analysis.

## 2. Materials and Methods 

### 2.1. Materials

**Figure 1:**
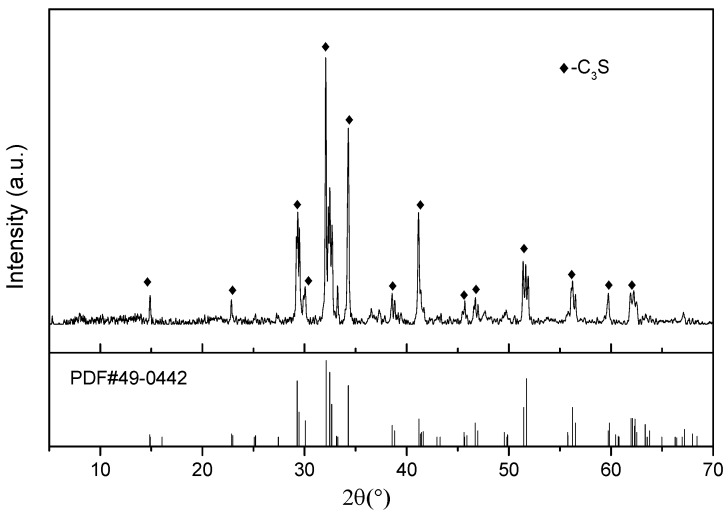
X-ray diffraction (XRD) patterns of tricalcium silicate (C_3_S).

The XRD pattern of the C_3_S used in this research is shown in Figure 1. According to the peak correspondence of PDF#49-0442, it can be seen that the sample tested was C_3_S crystal, and the sample was highly pure with almost no other crystal impurities. A GO dispersion (6.4 g/L, Laboratory synthesis) was also used in this experiment, along with pure CO_2_ supplied by Shente company (Shenzhen, China) and distilled water.

### 2.2. Synthesis of CaCO_3_ by the Carbonation Method

Carbonation was used in this experiment to synthesize CaCO_3_ [42]. Firstly, C_3_S was mixed with distilled water and different mass ratios of GO (0.2%, 1%, and 2%) in a three-necked flask. The mass ratio of C_3_S to water was set to 1/140. The mixture was stirred under sealed conditions for 24 h with a constant stirring rate of 300 rpm [43]. After 24 h of the hydration reaction, pure CO_2_ was introduced into the slurry through a tube at a speed of 100 ml/min. As the pH of the slurry decreased to 7, the passage of CO_2_ was stopped. The resulting precipitate was separated from the mother liquor by suction filtration and washed with distilled water. Finally, the precipitate was dried in an oven at 80 °C for 24 h. The hydration reaction of C_3_S produces calcium silicate hydrate (C-S-H) gel and the carbonation reaction can be represented by the following expressions [44]:

CO_2_ + H_2_O ↔ H_2_CO_3_ ↔ H^+^ + HCO_3_^−^ ↔ 2H^+^ + CO_3_^2−^ (1)

C_3_S + 6H_2_O → C-S-H + 3Ca(OH)_2_ (2)

Ca(OH)_2_ + 2H^+^ + CO_3_^2−^ → CaCO_3_ + 2H_2_O (3)

C-S-H + 2H^+^ + CO_3_^2−^ → CaCO_3_ + SiO_x_OH_x_ (4)

C-S-H + H^+^ + HCO_3_^−^ → CaCO_3_ + SiO_x_OH_x_ (5)

### 2.3. Characterization Methods

The morphology of the final CaCO_3_ products was observed using an environmental scanning electronic microscope (ESEM, Quanta TM 250 FEG, 20 KV, Hillsboro, OR, USA). The sample surface was coated with a thin layer of gold nanoparticles prior to ESEM testing. X-ray diffraction (XRD, D8 Advance, Bruker, Karlsruhe, Germany) was used to analyze the polymorph of the samples at a scanning rate of 2°/min from 10° to 70° with Cu Kα radiation (λ = 1.5405 Å) on a D max/RB diffractometer. The particle size distribution of the CaCO_3_ was measured by wide-angle, static-dynamic, synchronous laser scattering (Mastersizer 2000, Malvern, UK). All tests were carried out at room temperature.

## 3. Results and Discussion

### 3.1. Effect of GO on the Crystal Polymorph of CaCO_3_

**Figure 2:**
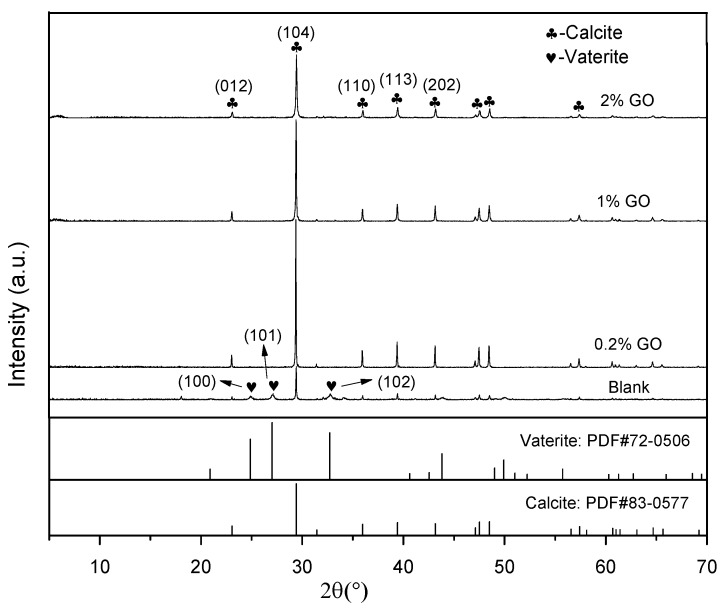
XRD patterns of calcium carbonate (CaCO_3_) crystals prepared with different content of graphene oxide (GO).

The CaCO_3_ crystals formed by carbonization of the C_3_S hydration products—with different mass ratios of GO—were measured by XRD. As shown in Figure 2, diffraction peaks of 2θ at 23.06°, 29.41°, 35.96°, 39.42°, and 43.17° corresponded with (012), (104), (110), (113), and (202) crystallographic planes of calcite, respectively, while peaks at 24.87°, 27.18° and 32.71° corresponded with (100), (101), and (102) crystallographic planes of vaterite [1]. In Figure 2, it can also be observed that the CaCO_3_ formed via the carbonization reaction without GO contained two crystals of calcite and vaterite. When GO was present, regardless of the amount, all of the CaCO_3_—obtained via the reaction of the C_3_S hydration product with CO_2_—was converted into calcite. Previous studies have shown that CaCO_3_ exhibits three polymorphic forms, namely calcite, aragonite and vaterite [45,46]. Among them, calcite is the most stable form in thermodynamics, while vaterite is the most unstable [47]. Combined with the XRD results, we can conclude that the addition of GO was able to promote the formation of calcite during the carbonation of the C_3_S hydration products. Yao et al. gave a similar conclusion when they used CaCl_2_ and (NH_4_)_2_CO_3_ (as sources of calcium) in conjunction with CO_2_ to prepare CaCO_3_ via a precipitation reaction [1]. Some researchers [1,28] believe that –COOH on the surface of GO can coordinate with Ca^2+^ ions in solution to provide a nucleation site for crystallization of CaCO_3_, thereby promoting crystallization of CaCO_3_. However, the present study shows an interesting phenomenon—that GO can still affect the crystallization of CaCO_3_ after participating in the C_3_S hydration for 24 h, indicating that the cement modified by GO is still likely to be affected by the presence of GO during carbonization. This phenomenon may be due to the linkage of GO to C-S-H [48], which affects the subsequent carbonization process.

### 3.2. Effect of GO on the Crystal Size of CaCO_3_

The Debye–Scherrer equation, as expressed in Equation (6), was used to analyze the crystal size of the samples [3].

(6) $$D=\frac{K\lambda }{\beta \cos\theta }$$

where: D is the size of grains in the direction perpendicular to the crystal plane; K is a constant (K = 0.89 when the particles are spherical or K = 0.943 when the particles are cubic); λ is the X-ray wavelength; β stands for the diffraction peak half-width when the particles are spherical, while β should be converted to radians, represented by β’, when the particles are cubic (Equation (7)); and θ is the diffraction angle of the X-ray. 

(7) β’=(β×π)/180

Table 1 shows the effect of GO dosage on the diffraction angle, peak height and crystal size of the calcite formed by the carbonation of C_3_S hydration products. As shown in Table 1, the diffraction angle of the calcite remained almost the same regardless of the GO content. This indicated that the addition of GO had no effect on the unit cell parameters of the calcite. The diffraction peak height of the (104) plane for the blank sample was 1860, and the corresponding crystal size of the calcite was 90.63 nm. However, compared with the blank sample, the diffraction peak intensity of the (104) plane for the calcite increased by 346.34%, 206.18%, and 90.38%, while the size of the calcite crystals decreased by 5.49%, 12.38%, and 24.61% when the amount of GO was set to 0.2%, 1%, and 2%, respectively. The peak height and crystal size of the calcite at (012), (110), (113) and (202) planes showed the same changes in regularity. A comparison of the diffraction peak heights and crystal sizes of each group showed that the addition of GO increased the peak height of the calcite while decreasing its crystal size. At the same time, with the increase of GO content, both the peak height and crystal size gradually decreased. Combined with the results of Figure 1, it can be concluded that the presence of GO promoted the conversion of CaCO_3_ to the most stable calcite crystal phase with high crystallinity. However, when the GO dosage was increased, the growth of the calcite crystal particles was somewhat suppressed, and thus the crystal grain size gradually reduced.

**Figure 3:**
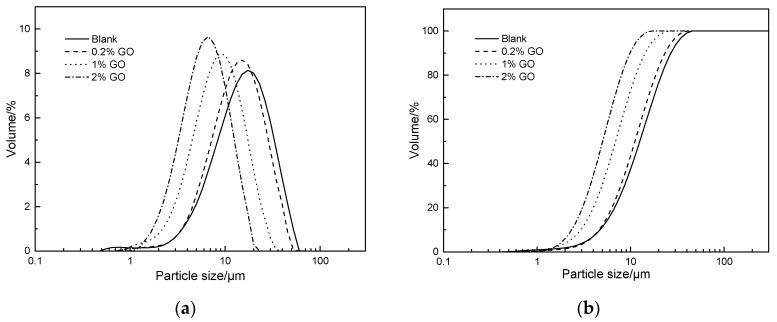
Particle size distribution of CaCO_3_ prepared with different dosages of GO: (**a**) Differential distribution, and (**b**) integral distribution.

The particle size distribution of the CaCO_3_ prepared with different mass ratios of GO were measured using a laser particle size analyzer. The differential and integral curves of the particle size distribution are shown in Figure 3. From this figure, it is obvious that both the differential and integral curves shifted to the left after the addition of GO, and that the shift amplitude increased with increasing GO dosage, indicating that the particle size of the CaCO_3_ particles formed by the carbonization reaction became smaller.

These results were then evaluated by the Rosin–Rammler distribution function, which is commonly used to simulate and analyze the particle size distribution of powders, such as dust and limestone [49]. The Rosin–Rammler distribution function is shown in Equation (8).

R(Dp) = 1 − exp[−(D_p_/D_e_)^n^] (8)

where: D_p_ is the corresponding particle diameter (μm); R(D_p_) is the cumulative percentage over size (%); De is a constant-related particle size; and the index, n, is a constant, which is related to the range of the particle size distribution. The larger the value of n, the narrower the distribution range of the particle diameter. The fitting results of the CaCO_3_ particle size distribution by the Rosin–Rammler function are displayed in Table 2.

As seen in Table 2, the n value of the CaCO_3_ particles formed when no GO was added in the preparation process was 1.847, with an average particle size of 15.705 μm. Interestingly, we found that the n value increased while De decreased with increasing GO dosage. When the amount of GO was gradually increased from 0.2% to 2%, the value of n continuously increased, whereas the value of De decreased significantly (17.21%, 39.26%, and 58.03% for 0.2%, 1%, and 2% GO content, respectively). This means that the particle size of CaCO_3_ gradually became smaller and the particle size distribution became more uniform with increasing GO content. It has previously been reported that the size of CaCO_3_ particles is highly dependent on both the nucleation rate and the crystal growth rate, and that groups on the surface of GO—such as the carboxyl groups—may provide nucleation sites for CaCO_3_ and thereby promote the formation of calcite [28,50]. The observed narrowing of the particle size distribution and the large reduction in particle size appears to support the notion that the addition of GO promotes the nucleation rate of CaCO_3_ while reducing its growth rate. These results are also consistent with the findings presented in Figure 2 and Table 1. However, it is worth considering that the carboxyl group on the surface of the GO—after participating in the hydration reaction—should have already captured the Ca^2+^. Therefore, it is unlikely to continue to be a nucleation site for CaCO_3_ crystallization during the carbonization process. There may be other mechanisms for the occurrence of this phenomenon, which requires further experimentation and analysis.

### 3.3. Effect of GO on the Morphology of CaCO_3_

**Figure 4:**
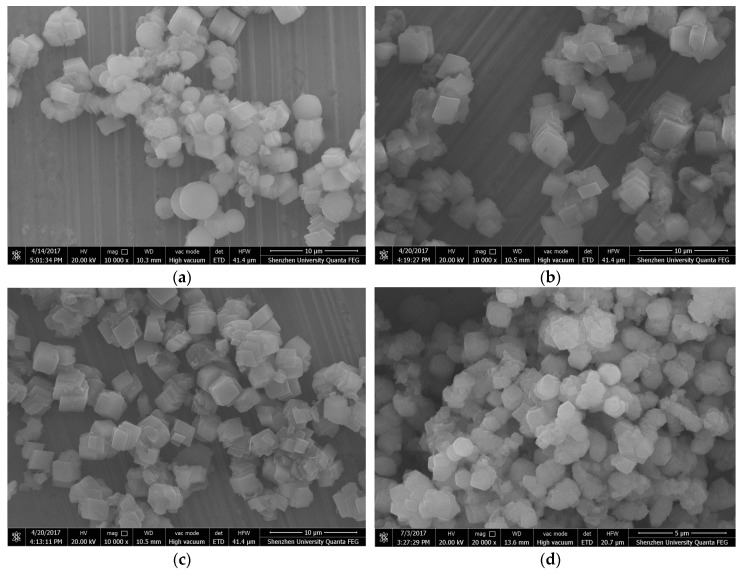
Environmental scanning electronic microscope (ESEM) images of CaCO_3_ crystals prepared with different mass ratios of GO: (**a**) No GO, (**b**) 0.2% GO, (**c**) 1% GO, and (**d**) 2% GO.

Figure 4 presents ESEM images of the CaCO_3_ prepared with different contents of GO. As depicted in Figure 4a, the CaCO_3_ crystals were found to be cubic and spherical in shape when no GO was added into the mixture. According to the XRD results in Figure 2, the cubic-shaped particles are considered to be calcite while the spherical-shaped particles are vaterite. As shown in Figure 4b–d, after the addition of GO, the CaCO_3_ generated via carbonation were all square-shaped calcites. From these results, it can be concluded that the addition of GO created cubic-shaped CaCO_3_, and that changes in GO content did not affect the shape of the CaCO_3_. This is consistent with the findings shown in Figure 2.

## 4. Conclusions and Recommendations

The effect of GO on the crystal phase and crystal size of CaCO_3_ during the carbonation of C_3_S hydration products was investigated. From the results of XRD and SEM analysis, we concluded that the presence of GO promoted the conversion of CaCO_3_ to a crystal phase with higher thermal stability than the control—i.e., to a calcite crystal phase. However, an increase in the amount of GO had a certain inhibitory effect on the growth of the calcite crystal particles. From the test results, the crystal size of calcite became the largest when the lowest amount of GO was added. After participating in C_3_S hydration for 24 h, GO still had an effect on the polymorphism and crystal size of CaCO_3_, but the mechanism remains uncertain and further studies are needed. Although the polymorphism and crystal size of CaCO_3_ are discussed, the mechanical properties—such as elastic modulus and hardness value—have not been fully studied, and thus will become the focus of future research. 

## Figures and Tables

**Table 1:** The diffraction angle, peak height and crystal size of the calcite prepared with different content of GO.

h, k, l	GO%	2θ(°)	Peak Height	Crystal Size (nm)
012	0	23.031	178	92.38
	0.2	23.053	697	87.62
	1	23.055	549	81.72
	2	23.055	225	60.71
104	0	29.369	1860	90.63
	0.2	29.399	8302	85.65
	1	29.400	5695	79.41
	2	29.400	3541	68.33
110	0	35.936	227	84.43
	0.2	35.968	954	79.34
	1	35.973	674	74.83
	2	35.973	417	67.35
113	0	39.369	332	93.11
	0.2	39.408	1441	83.44
	1	39.411	964	80.41
	2	39.411	588	59.37
202	0	43.116	265	93.28
	0.2	43.157	1221	89.72
	1	43.162	875	81.41
	2	43.162	501	60.11

**Table 2:** Fitting results of CaCO_3_ particle size distribution.

GO%	n Value	De Value (μm)
0	1.847	15.705
0.2	1.999	13.002
1	2.159	9.540
2	2.256	6.591

## Abbreviations

GO	Graphene oxide
CaCO_3_	Calcium carbonate
C_3_S	Tricalcium silicate (3CaO·SiO_2_)
C-S-H	Calcium silicate hydrate (3CaO·SiO_2_ 3H_2_O)
CO_2_	Carbon dioxide
ESEM	Environmental scanning electronic microscope
XRD	X-ray diffraction
CaCl_2_	Anhydrous calcium chloride
AFt	Ettringite
AFm	Monosulfate
